# Betapapillomavirus natural history and co-detection with alphapapillomavirus in cervical samples of adult women

**DOI:** 10.1002/jmv.29288

**Published:** 2023-12

**Authors:** Talía Malagón, Aline Lopes Ribeiro, Emily Montosa Nunes, Tarik Gheit, Mariam El-Zein, Luisa L. Villa, Eduardo L. Franco, Laura Sichero

**Affiliations:** 1 Gerald Bronfman Department of Oncology, Division of Cancer Epidemiology, McGill University, Montréal, Canada; 2 St Mary's Research Centre, Montreal West Island Integrated University Health and Social Services Centre, Montréal, Canada; 3 Center for Translational Research in Oncology, Instituto do Cancer do Estado de Sao Paulo ICESP: Hospital das Clínicas da Faculdade de Medicina da Universidade de São Paulo FMUSP HC, São Paulo, Brazil; 4 Comprehensive Center for Precision Oncology, Universidade de Sao Paulo, São Paulo, Brazil; 5 Epigenomics and Mechanisms Branch, International Agency for Cancer Research (IARC), Lyon, France; 6 Department of Radiology and Oncology, Faculdade de Medicina, Universidade de São Paulo, São Paulo, Brazil

**Keywords:** epidemiology, human papillomavirus, research and analysis methods, sexually transmitted disease

## Abstract

Human papillomaviruses (HPV) of the genus *Betapapillomavirus* can infect both cutaneous and mucosal sites, but research on their natural history at mucosal sites remains scarce. We examined the risk factors and co-detection patterns of HPVs of the *Betapapillomavirus* and *Alphapapillomavirus* genera in cervical samples of the Ludwig-McGill cohort study. We assessed a subset of 505 women from the Ludwig-McGill cohort study from São Paulo, Brazil. Cervical samples over the first year of follow-up were tested for DNA of over 40 alphapapillomavirus types and 43 betapapillomavirus types using a type-specific multiplex genotyping polymerase chain reaction assay. We assessed the risk factors for prevalent and incident betapapillomavirus type detection, and whether types were detected more frequently together than expected assuming independence using permutation tests, logistic regression, and Cox regression. We observed significant within-genus clustering but not cross-genus clustering. Multiple betapapillomavirus types were co-detected in the same sample 2.24 (95% confidence interval [CI]: 1.65–3.29) times more frequently than expected. Conversely, co-detections of alphapapillomavirus and betapapillomavirus types in the same sample occurred only 0.64 (95% CI: 0.51–0.83) times as often as expected under independence. In prospective analyses, positivity to one HPV genus was associated with a nonsignificant lower incidence of detection of types in the other genus. Lifetime number of sex partners and new sex partner acquisition were associated with lower risks of prevalent and incident betapapillomavirus detection. Betapapillomaviruses are commonly found in the cervicovaginal tract. Results suggest potentially different mechanisms of transmission for betapapillomavirus genital infections other than vaginal sex.

## 1 ∣ INTRODUCTION

Human papillomaviruses (HPV) constitute several genera. The vast majority of previous research on HPV has focused on the genus *Alphapapillomavirus*, whose types infect the mucosal epithelium and are an established cause of anogenital and some oropharyngeal cancers.^1^ HPVs of the genus *Betapapillomavirus* normally infect the cutaneous epithelium.^2^ Comparatively much less research has been done on the epidemiology of infections with betapapillomaviruses. However, growing evidence suggests that betapapillomaviruses may play a role in the development of skin cancers, and may be associated with the risk of a subset of head and neck cancers.^1,3-5^ More research on the incidence, transmission, and risk factors for betapapillomavirus infection is warranted.

While betapapillomaviruses were first isolated from the skin and are widely considered to prefer infecting the cutaneous epithelium, there is substantial evidence that they are also able to infect the mucosal epithelium.^4,6^ The detection of betapapillomaviruses in oral and genital samples suggests they are able to infect these sites along with alphapapillomaviruses.^7-12^ There is, therefore, the potential for biological interactions between HPV types of different genera. While co-detection patterns and the potential for interactions between HPV types within the alphapapillomavirus genus has been the topic of substantial previous research,^13-16^ there is scant research on cross-genus co-detection patterns and interactions. Biological interactions and co-detection patterns between different HPV types are important to study from a public health perspective due to the risk of genotype replacement following widespread implementation of HPV vaccination programs.^17^ Although there is currently little evidence that genotype replacement has occurred for alphapapillomaviruses,^18^ the potential for interactions with other HPV genera has not been studied.

We had previously found a fairly high prevalence of betapapillomaviruses in a limited number of cervicovaginal samples of the Ludwig-McGill cohort study of HPV natural history.^19^ While we examined a number of putative risk factors, the only variable that was associated with betapapillomavirus prevalence in univariate analyses was lifetime number of sex partners. Curiously, we found a lower betapapillomavirus prevalence among women with four or more lifetime sex partners in univariate analyses; it was, however, unclear whether this result represented a true causal effect rather than a statistical fluke or the result of confounding bias. We also did not find that alphapapillomaviruses and betapapillomavirus co-detection patterns deviated from expectations under independence. However, this previous analysis was underpowered to assess the co-detection patterns of alphapapillomavirus and betapapillomavirus, so we were unable to draw firm conclusions about whether different genera cluster together or not. We previously also only looked at prevalent but not incident co-detections.

The objective of the current study was to expand these previous analyses using a larger number of samples to increase our statistical power to (i) calculate incidence rates of betapapillomavirus detection, (ii) examine whether there is evidence of biological interactions between alphapapillomaviruses and betapapillomaviruses, and (iii) assess risk factors for betapapillomavirus genital prevalence and incidence in multivariable analyses to adjust for potential confounders.

## 2 ∣ METHODS

### 2.1 ∣ Study population

The Ludwig-McGill study cohort study of HPV natural history recruited women at maternal health clinics in São Paulo, Brazil, between 1993 and 1997. Details of the study design have been published elsewhere.^20^ Eligible women had to be between 18 and 60 years old, permanent residents of São Paulo, not currently pregnant, with an intact uterus, and with no history of treatment for cervical disease. During the first year, women were followed-up every 4 months; at each visit, they provided cervical specimens for HPV genotyping and underwent an interview administered by study nurses collecting data on sociodemographic information, reproductive health, sexual behaviors, smoking, and diet. Women provided signed informed consent to study participation. The study was approved by the ethical review boards of all participating institutions: McGill University, Montreal, Canada; University of Toronto, Ontario, Canada; and the Ludwig Institute for Cancer Research and the Hospital Maternidade Vila Nova Cachoeirinha, both in São Paulo, Brazil.

The current analysis is restricted to a random subset of 505 women sampled from the full cohort who had a follow-up visit within 10 days of the 1-year follow-up date, had valid samples (either β-globin or HPV-positive samples) at the baseline and 1-year visits, and who had complete questionnaire and HPV genotyping data. Our previous publication details the selection process, baseline characteristics, and the betapapillomavirus prevalence at baseline (first visit) and 1-year (fourth visit) in this subset of women.^19^ For the current study, we extended genotyping to the 4-month (second visit) and 8-month (third visit) samples collected from these women to measure betapapillomavirus incidence rates over the first year of follow-up and increase the number of observations for analyses.

### 2.2 ∣ HPV genotyping

Exfoliated cervical cells were digested with 100 μg/mL proteinase K for 3–18 h at 55°C, and DNA was obtained by spin column chromatography. Samples were tested for alphapapillomaviruses by polymerase chain reaction (PCR) amplification using MY09/11 or PGMY09/11 generic primers, and genotyped using hybridization with HPV type-specific oligonucleotide probes or restriction fragment length polymorphism analysis. This methodology allowed the identification of potentially more than 40 genital alphapapillomavirus genus types from the following species: α-1: HPVs 32, 42; α-3: HPVs 61, 62, 72, 81, 83, 84, 89; α-4: HPV57; α-5: HPVs 26, 51, 69, 82; α-6: HPVs 53, 56, 66; α-7: HPVs 18, 39, 45, 59, 68, 70; α-8: HPV40; α-9: HPVs 16, 31, 33, 35, 52, 58, 67; α-10: HPVs 6, 11, 44; α-11: HPVs 34, 73; α-13: HPV54; and α-14: HPV71.^21^

The presence of betapapillomaviruses was assessed by a type-specific, multiplex genotyping PCR assay followed by genotyping via a bead-based Luminex technology.^22^ This assay distinguishes 43 betapapillomavirus genus types from the following species: β-1: HPVs 5, 8, 12, 14, 19, 20, 21, 24, 25, 36, 47, 93, 98, 99, 105, 118 124, 143; β-2: HPVs 9, 15, 17, 22, 23, 37, 38, 80, 100, 104, 107, 110, 111, 113, 120, 122, 145, 151; β-3: HPVs 49, 75, 76, 115; β-4: HPV92; and β5: HPVs 96, 150. In this assay, results are expressed as the median fluorescence intensity (MFI) of at least 100 beads per bead set. For each probe, the MFI values obtained when no PCR product was added to the hybridization mixture were considered the background values. Different bead preparations impact MFI values; because the samples from visits 2 and 3 were tested using a different preparation of beads than the samples from visits 1 and 4, a different MFI cutoff value was used for these visits. Based on MFI values from positive and negative controls reads, cutoffs were computed by adding 20 MFI to 1.1X the median background values in samples obtained in visits 1 and 4^20^; and by adding 50 MFI to 1.1X the median background values for samples obtained in visits 2 and 3, with the exception of HPV100 for which the cutoff was computed by adding 100 MFI to 1.1X the median background values for visits 2 and 3.

### 2.3 ∣ Statistical analysis

All analyses were type-specific, with HPV type as the unit of observation. The prevalences of betapapillomaviruses were summed across the four visits to derive a time-averaged measure of prevalence over the first year of the study. The incidence rates of betapapillomaviruses were calculated in women who were negative for that specific betapapillomavirus type at the previous visit, with the denominator being the time between visits. We pooled results by summing observations over all HPV types.

**FIGURE 1:**
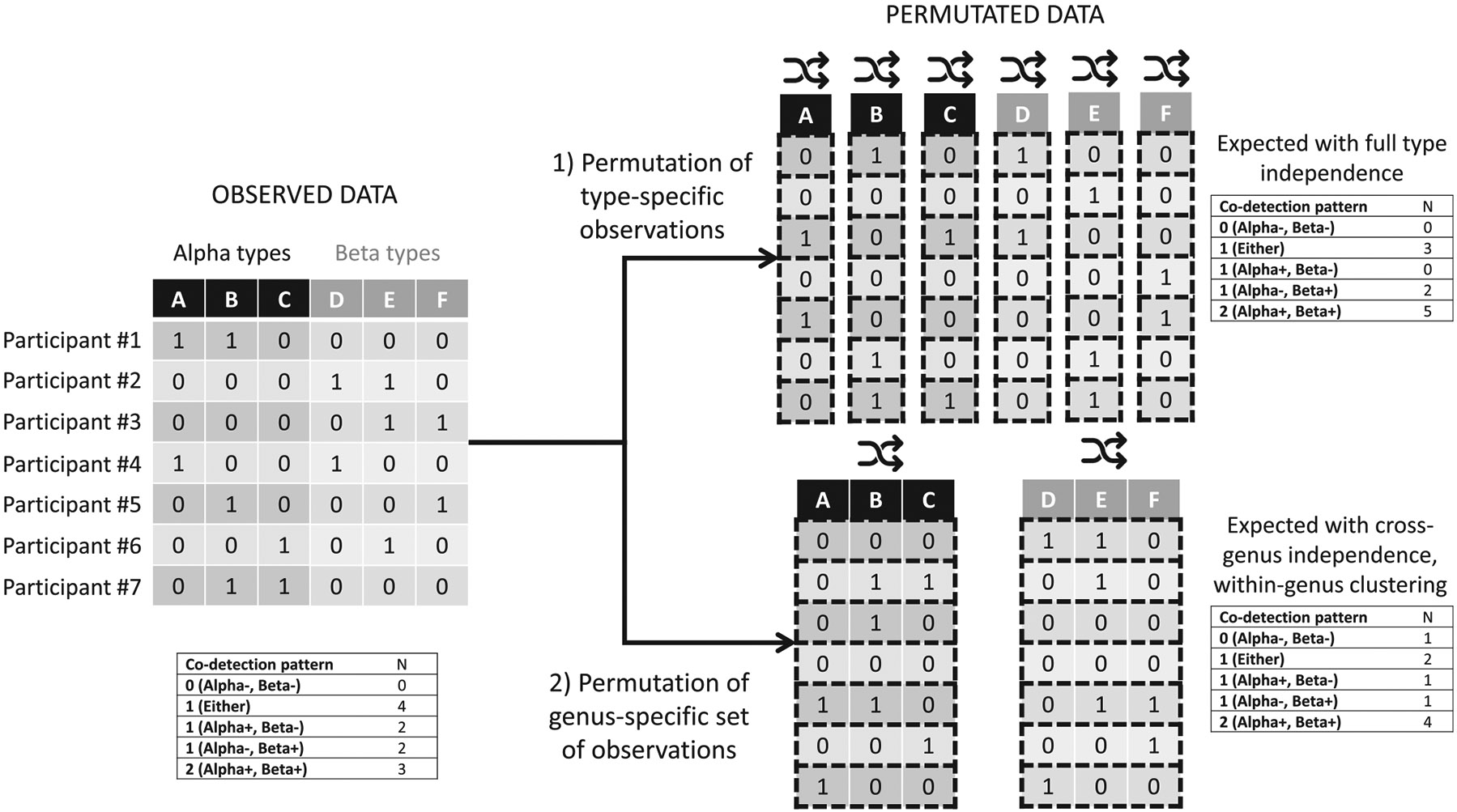
Graphical representation of permutation tests. The first test permutates the data for each human papillomavirus (HPV) type individually and derives the expected distribution of HPV positivity assuming full independence for all HPV types. The second test permutates the data for the set of HPV types within each genus and derives the expected distribution of HPV positivity assuming independence between genera while maintaining the clustering of types within a genus.

To assess whether betapapillomaviruses and alphapapillomaviruses occur more frequently together than expected, we used permutation tests. Permutation tests work by performing rearrangements of the observed data without replacement (permutations).^23^ The permutations allow deriving an expected distribution of betapapillomaviruses and alphapapillomaviruses under the assumption that infections are independently distributed across individuals. Because there was evidence of within-genus clustering, we implemented two versions of the permutation test (Figure 1). The first test assumed full independence of all HPV types; in this case, the results for each individual HPV type are permutated across participants, with each HPV type resampled separately. This first test will give the expected distribution of type-specific detections assuming all HPV types are fully independent, both across and within genera. The second test assumed independence between papillomavirus genera but not within genus; in this case, the set of results from all HPV types within a genus from the same person are permutated across participants, with each genus resampled separately. This second test will give the expected distribution of type-specific detections assuming that alphapapillomaviruses are independent from betapapillomaviruses and vice-versa, but accounting for the tendency of HPV types from the same genus to cluster together. We performed 2500 permutation resamples for both tests. We divided the observed number of co-detections by the mean number of co-detections in the permutation resamples to obtain observed over expected values. The 95% confidence intervals (CIs) were obtained using the 2.5–97.5 percentiles of the permutation resample distribution.

To assess the cross-sectional association between alphapapillomavirus and betapapillomavirus prevalence, we calculated odds ratios (OR) using random effects logistic regression models, with a woman-level random intercept. To assess the prospective association between alphapapillomavirus and betapapillomavirus incidence, we calculated hazard ratios (HR) using Cox proportional hazard regression models, using the robust sandwich estimate of the covariance to account for multiple HPV types per woman.^24^ All models were fitted separately for alphapapillomavirus and betapapillomavirus types and used the HPV type as the unit of analysis. To assess interactions between genera, the models included as predictors whether the woman was positive for any HPV type of a different genus at either the same visit sample (logistic regressions) or the previous visit sample (Cox regressions). Multivariable models were adjusted for age and sexual behaviors reported since the previous visit. Sexual behavior variables were included as a priori predictors because our previous publication had found that lifetime number of sex partners might be inversely associated with betapapillomavirus prevalence.^19^

## 3 ∣ RESULTS

All 505 women contributed four study visits each over the 1st year; however, there were missing samples for eight women (1.6%) at the 4-month visit and for 12 women (2.4%) at the 8-month visit, for a final total of 2000 study visits with samples. All available samples were valid (positive for either β-globin or HPV). Baseline characteristics of the subcohort have previously been reported^19^; the mean age was 33.5 years (range: 18–57) and most women were married (49%) or living as married (33%). There were 281/2000 (14%) samples positive for any betapapillomavirus across all study visits. The prevalence and incidence of individual betapapillomaviruses are presented in Table 1. The most prevalent and incident types were HPV38, HPV21, HPV5, HPV22, and HPV8, respectively; these were the only types with a time-averaged prevalence above 1%.

We observed significant clustering within HPV genera (Table 2). Over the four visits, we observed 44 samples positive for two or more alphapapillomavirus types, and 56 samples positive for two or more betapapillomavirus types. This represents respectively 1.74 (95% CI 1.26–2.44) times more alphapapillomavirus type co-detections than expected and 2.24 (95% CI 1.65–3.29) times more betapapillomavirus type co-detections than expected when assuming independence of types. The betapapillomavirus types most often found in co-detection with others were also the most commonly detected types (HPV38, HPV21, HPV5, HPV22, and HPV8).

Conversely, there were fewer than expected co-detections of alphapapillomaviruses and betapapillomaviruses (Table 2). Over the four visits, we observed 33 samples positive for both an alphapapillomavirus and a betapapillomavirus type together. This represents 0.64 (95% CI 0.51–0.83) times fewer cross-genus co-detections than expected when assuming independence of all types. After accounting for the within-genus expected clustering of both genera, this represented 0.80 (95% CI 0.62–1.06) times fewer cross-genus detections than expected when assuming cross-genus independence.

Cross-sectional and prospective associations between betapapillomavirus positivity, alphapapillomavirus positivity, and sexual risk factors are presented in Tables 3 and 4. Samples were somewhat less likely to be positive for alphapapillomavirus types if a betapapillomavirus type was present (OR 0.73, 95% CI 0.50–1.07). They were also somewhat less likely to be positive for betapapillomavirus types if an alphapapillomavirus type was present (OR 0.79, 95% CI 0.56–1.12), but neither of these associations were significant. Women were also less likely to become newly positive for alphapapillomavirus types if a betapapillomavirus type was present at the previous visit (HR 0.84, 95% CI 0.49–1.43) and were less likely to become newly positive for betapapillomavirus types if an alphapapillomavirus type was present at the previous visit (HR 0.72, 95% CI 0.45–1.15), but these associations were also not significant. Alphapapillomavirus prevalence and incidence were strongly associated with age, increasing lifetime number of sex partners, and having new sex partners in the previous interval. Conversely, betapapillomavirus prevalence and incidence were not associated with age, and were inversely correlated with the number of lifetime sex partners. While having a new sex partner in the previous interval was not associated with prevalence or incidence, having sex with any partner in the previous interval was associated with a higher incidence of betapapillomavirus (HR 1.56, 95% CI 1.00–2.46). Multivariable adjustment did not change these associations.

## 4 ∣ DISCUSSION

We found that alphapapillomavirus and betapapillomavirus types were detected together significantly less often than expected under independence of HPV types. Some of this was due to intragenus clustering: both alphapapillomavirus and betapapillomavirus types are more likely to be detected with other types from the same genus. This clustering is likely due to shared common transmission routes with closely related types. There were still fewer than expected co-detections of alphapapillomavirus and betapapillomavirus types than expected after adjusting for intragenus clustering, though CIs could not exclude independence.

A substantial amount of previous research has shown that alphapapillomaviruses tend to cluster together; individuals who are positive for one alphapapillomavirus type are at a significantly higher risk of becoming positive for other alphapapillomavirus types.^13-16^ Presumably a large part of this association reflects shared risk factors across all alphapapillomavirus types, the most important being a common sexual transmission route as well as host-level susceptibility to infection. Therefore, it is unsurprising that we also found betapapillomaviruses tend to cluster together. This presumably also reflects a shared common transmission route across all betapapillomavirus types. However, what is much more surprising is the definite lack of clustering between alphapapillomaviruses and betapapillomaviruses. While the CIs of many of the measures of association include the null value expected under independence (1.0), all previous evidence^13-16^ supports that independence between HPV types is not the expectation and that the expected clustering of HPVs of the alphapapillomavirus genus is of the order of OR/HRs of at least 1.5–3.0. Therefore, while the CIs include ORs and HRs of 1.0, we consider that this is a highly unusual result. We examine below two potential hypotheses for why alphapapillomaviruses and betapapillomaviruses may be less likely to be found together than expected.

The first hypothesis relates to potential biological interactions between genera. This could arise, for example, if infection with a first genus induces cross-immunity to subsequent infection with the other genus, or if both genera occupy the same ecological niche and infection with one genus leads to competitive exclusion of the other genus.^17^ We believe this hypothesis to be implausible for several reasons. HPV antibodies induced by natural infection tend to be type-specific,^25^ so it is unlikely that infection with types from one HPV genera would generate strong neutralizing cross-protection against infection with types from another HPV genera. Alphapapillomaviruses and betapapillomaviruses are also believed to have distinct tissue tropisms with preferred anatomical sites of infection.^26^ While alphapapillomaviruses have evolved to infect the mucosal epithelium, betapapillomaviruses have evolved to infect the cutaneous epithelium.^2^ Nonetheless, both genera are able to infect epithelial sites outside of their preferred trophic niche,^6,8,27^ so, it is not impossible they could have overlapping niches leading to competitive exclusion. If this were the case, we would expect alphapapillomavirus types to be more likely to exclude cervicovaginal infections with betapapillomavirus types than the reverse due to their mucosal tropism. However, we did not find that alphapapillomavirus positivity reduced incident betapapillomavirus more than the reverse, so the hypothesis of competitive exclusion does not seem to be supported.

The second hypothesis is that the negative associations between genital alphapapillomaviruses and betapapillomaviruses could reflect independent or inversely correlated transmission mechanisms. Alphapapillomaviruses are sexually transmitted infections, with major risk factors including lifetime number of sex partners and new sex partners.^28^ Betapapillomaviruses are believed to be transmitted through skin-to-skin contacts,^2^ which include but are not restricted to sexual contacts. Interestingly, we found that lifetime and new sex partners were not risk factors for betapapillomavirus incidence, but having any sex during the previous interval was a risk factor. Most of the women who reported having sex in our study were having sex with a regular partner rather than a new one, and women who were married or living with their partner reported higher frequencies of sexual contacts than single or separated women. It could be that, in this study, having sex is an indicator for being in an ongoing intimate relationship with more skin-to-skin contacts, which include but may not be restricted to sex. It is also possible that betapapillomavirus detections may be depositions from recent sex rather than true infections, as the penile epithelium has been found to have a high betapapillomavirus prevalence.^9^ However, a previous study did not find that vaginal sex within the last 24 h increased genital betapapillomavirus concordance between heterosexual partners.^8^ Nevertheless, that study and others have found that concordance was higher within than between couples, suggesting some form of transmission through intimate contact.^8,29^ Fingernails could be a potential reservoir for transmission and autoinoculation in the case of oral betapapillomavirus infections.^11^ We believe it is plausible that the negative association observed between betapapillomavirus and alphapapillomavirus types could partly reflect confounding due to inversely correlated transmission mechanisms, as women in our study who were having more regular sex and intimate skin-to-skin contact were women with fewer lifetime sexual partners or new sex partners. However, our adjustment for these sexual behaviors in multivariable models did not completely attenuate the negative association between alphapapillomaviruses and betapapillomaviruses. There could, therefore, remain some unmeasured confounding transmission risk factors explaining the negative association between betapapillomaviruses and alphapapillomaviruses.

The most detected betapapillomavirus types in cervicovaginal samples were HPV38, HPV5, HPV21, HPV22, and HPV8, respectively. Moscicki et al. also found that HPV38 was the most commonly detected betapapillomavirus type in cervicovaginal samples.^8^ HPV38 is also one of the most commonly detected types in women's oral samples,^11^ and among the most common betapapillomavirus types in men's genital samples.^9^ Genital HPV21 and HPV22 positivity were also common in male genital samples.^9^ HPV5 and HPV8 were conversely not among the most commonly reported detected types in male genital samples.^9^ HPV5 and HPV8 are the betapapillomavirus types which have been isolated from patients with epidermodysplasia verruciformis (EV) and are deemed to be possibly carcinogenic for individuals with EV by the International Agency for Research on Cancer.^1,30^ Oral HPV5 has also been associated with the risk of some head and neck cancers.^3^

By increasing the numbers of samples tested for betapapillomavirus, we were able to expand on our previous results which only included two observations per woman.^19^ The current study confirms our initial surprising findings that lifetime number of sex partners was inversely correlated with betapapillomavirus prevalence, and we propose above a potential explanation for this finding based on a multivariable analysis of sexual risk factors. However, this finding might reflect confounding from sexual behaviors that is specific to our study population, as studies of oral and penile betapapillomavirus infections have not found that number of lifetime sex partners was associated with betapapillomavirus prevalence at these sites.^9,11,12,31^ We are unaware of any other studies that have looked at risk factors for cervicovaginal betapapillomavirus prevalence. Due to the larger sample size, we were able to calculate incidence rates of betapapillomavirus positivity, and also detect lower than expected prevalent and incident co-detections of alphapapillomavirus and betapapillomavirus types which were not evident in our previous analysis which had fewer observations. The analysis method we used in this paper (permutation tests) allowed us to derive CIs for observed/expected ratios which are considerably tighter than in our previous analysis, and which allow us to conclude with high certainty that alphapapillomaviruses and betapapillomaviruses do not cluster together and may potentially be less likely to be found together than expected. Our results would need to be confirmed in other studies of alphapapillomavirus and betapapillomavirus co-detection to verify whether they represent a more general trend across populations.

In conclusion, we found fewer prevalent and incident co-detections of alphapapillomaviruses and betapapillomaviruses than expected if HPV types belonging to these genera were transmitted independently. It is not clear whether these highly unusual findings reflect potential biological interactions between HPV genera, or are the result of inversely correlated transmission mechanisms, or simply represent a chance result. These findings need to be confirmed in other populations. This would help further elucidate the biology of betapapillomaviruses, a genus for which there is growing evidence of a role in carcinogenesis.

## Figures and Tables

**TABLE 1:** Betapapillomavirus time-averaged prevalences and incidence rates, overall and by type, age, and lifetime number of sex partners.

Variables		Cross-sectional		Prospective	
	
		*n/N*	Time-averaged prevalence (%)^a^	Events (*n*)	Incidence rate (/100 person-years)
Total	All (summed across types)	352/86 000	0.41	257	1.2
HPV type	HPV5	33/2000	1.65	28	5.6
	HPV8	25/2000	1.25	19	3.8
	HPV9	2/2000	0.10	1	0.2
	HPV12	11/2000	0.55	9	1.8
	HPV14	4/2000	0.20	4	0.8
	HPV15	5/2000	0.25	2	0.4
	HPV17	7/2000	0.35	5	1.0
	HPV19	4/2000	0.20	3	0.6
	HPV20	0/2000	0.00	0	0.0
	HPV21	40/2000	2.00	19	3.8
	HPV22	30/2000	1.50	22	4.4
	HPV23	8/2000	0.40	7	1.4
	HPV24	18/2000	0.90	15	3.0
	HPV25	0/2000	0.00	0	0.0
	HPV36	12/2000	0.60	12	2.4
	HPV37	0/2000	0.00	0	0.0
	HPV38	46/2000	2.30	31	6.3
	HPV47	6/2000	0.30	6	1.2
	HPV49	11/2000	0.55	10	2.0
	HPV75	0/2000	0.00	0	0.0
	HPV76	13/2000	0.65	10	2.0
	HPV80	0/2000	0.00	0	0.0
	HPV92	1/2000	0.05	1	0.2
	HPV93	0/2000	0.00	0	0.0
	HPV96	3/2000	0.15	2	0.4
	HPV98	0/2000	0.00	0	0.0
	HPV99	0/2000	0.00	0	0.0
	HPV100	13/2000	0.65	10	2.0
	HPV104	0/2000	0.00	0	0.0
	HPV105	3/2000	0.15	2	0.4
	HPV107	2/2000	0.10	2	0.4
	HPV110	11/2000	0.55	8	1.6
	HPV111	16/2000	0.80	6	1.2
	HPV113	1/2000	0.05	0	0.0
	HPV115	0/2000	0.00	0	0.0
	HPV118	0/2000	0.00	0	0.0
	HPV120	15/2000	0.75	13	2.6
	HPV122	8/2000	0.40	8	1.6
	HPV124	2/2000	0.10	1	0.2
	HPV143	0/2000	0.00	0	0.0
	HPV145	1/2000	0.05	1	0.2
	HPV150	0/2000	0.00	0	0.0
	HPV151	1/2000	0.05	0	0.0

Abbreviation: HPV, human papillomavirus.

a Time-averaged over four study visits.

**TABLE 2:** Observed versus expected number of samples positive for alphapapillomavirus and betapapillomavirus types based on permutation tests.

Within-genus clustering	Observed (*n*)	Expected^a^ (*n*)	Observed/ expected^a^	95% CI
Alphapapillomavirus, number of types detected in sample		Expected with total type independence		
0	1718	1693	1.01	1.01–1.02
1	257	299	0.86	0.81–0.91
2+	44	26	1.74	1.26–2.44
Betapapillomavirus, number of types detected in sample		Expected with total type independence		
0	1719	1675	1.03	1.02–1.03
1	225	299	0.75	0.71–0.80
2+	56	26	2.24	1.65–3.29
Cross-genus clustering				
Number of genera detected in sample		Expected with total type independence		
0 (Alpha−, Beta−)	1456	1405	1.04	1.02–1.05
1 (Either)	511	543	0.94	0.90–0.99
1 (Alpha+, Beta−)	263	270	0.97	0.93–1.03
1 (Alpha−, Beta+)	248	273	0.91	0.86–0.96
2 (Alpha+, Beta+)	33	52	0.64	0.51–0.83
Number of genera detected in sample		Expected with within-genus clustering		
0 (Alpha−, Beta−)	1456	1463	1.00	0.99–1.00
1 (Either)	511	495	1.03	0.99–1.08
1 (Alpha+, Beta−)	263	256	1.03	0.99–1.07
1 (Alpha−, Beta+)	248	239	1.04	0.99–1.09
2 (Alpha+, Beta+)	33	42	0.80	0.62–1.06

Note: CI, based on 2.5th and 97.5th percentiles of 2500 permutations.

Abbreviation: CI, confidence interval.

a Mean of 2500 permutations.

**TABLE 3:** Cross-sectional association between alphapapillomavirus and betapapillomavirus type-specific positivity (summed overall types within genera), age, and lifetime number of sex partners.

Risk factor	Alphapapillomavirus prevalence, summed overall types						Betapapillomavirus prevalence, summed overall types					
	
	n/N	Time-averaged prevalence (%)^a^	OR	95% CI	ORadj^b^	95% CI	*n/N*	Time-averaged prevalence (%)^a^	OR	95% CI	ORadj^b^	95% CI
Positivity to other HPV genus^c^												
Positive any	34/10 678	0.32	0.73	0.50–1.07	0.75	0.51–1.11	42/12 728	0.33	0.79	0.56–1.12	0.83	0.58–1.18
Negative any	314/65 322	0.48	1.00	Ref	1.00	Ref	310/73 272	0.42	1.00	Ref	1.00	Ref
Age years												
<25	105/13 832	0.76	1.00	Ref	1.00	Ref	67/15 566	0.43	1.00	Ref	1.00	Ref
25–34	123/28 614	0.43	0.48	0.29–0.80	0.49	0.30–0.81	138/32 121	0.43	0.96	0.68–1.36	0.95	0.67–1.35
35–44	93/22 838	0.41	0.44	0.25–0.76	0.46	0.27–0.78	91/25 542	0.36	0.79	0.55–1.15	0.79	0.55–1.16
≥45	32/11 438	0.28	0.32	0.16–0.63	0.31	0.15–0.61	56/12 771	0.44	1.01	0.66–1.55	1.02	0.66–1.56
Lifetime number of sex partners												
0–1	102/35 188	0.29	1.00	Ref	1.00	Ref	179/39 388	0.45	1.00	Ref	1.00	Ref
2–3	149/27 018	0.55	1.65	1.13–2.42	1.62	1.10–2.37	122/30 401	0.40	0.89	0.68–1.16	0.90	0.68–1.18
4+	102/14 478	0.70	2.17	1.38–3.42	2.13	1.36–3.36	50/16 168	0.31	0.68	0.47–0.97	0.71	0.49–1.03
Any sex in last interval												
No	47/7600	0.62	1.00	Ref	1.00	Ref	30/8514	0.35	1.00	Ref	1.00	Ref
Yes	306/69 122	0.44	0.65	0.40–1.05	0.58	0.35–0.94	322/77 486	0.42	1.21	0.80–1.84	1.21	0.78–1.87
New sex partner in last interval												
No	331/74 480	0.44	1.00	Ref	1.00	Ref	343/83 505	0.41	1.00	Ref	1.00	Ref
Yes	22/2242	0.98	1.10	0.62–1.95	1.11	0.62–1.98	9/2494	0.36	0.89	0.44–1.80	1.01	0.49–2.07

Abbreviations: CI, confidence interval; HPV, human papillomaviruses; OR, odds ratio; ORadj, adjusted odds ratio; Ref, reference level.

a Time-averaged over four study visits.

b Adjusted for positivity to the other HPV genus, age, lifetime number of sex partners, any sex in last interval, and new sex partner in last interval.

c Positive for any type of the other HPV genus at same visit; corresponds to betapapillomavirus positivity for columns where alphapapillomavirus prevalence is the outcome, and alphapapillomavirus positivity for columns where betapapillomavirus prevalence is the outcome.

**TABLE 4:** Prospective association between alphapapillomavirus and betapapillomavirus type-specific positivity (summed overall types within genera), age, and lifetime number of sex partners.

Risk factor	Alphapapillomavirus incidence, summed overall types						Betapapillomavirus incidence, summed overall types					
	
	Events (*n*)	Incidence rate (/100 person-years)	HR	95% CI	HRadj^a^	95% CI	Events (n)	Incidence rate (/100 person-years)	HR	95% CI	HRadj^a^	95% CI
Positivity to other HPV genus^b^												
Positive any	18	0.7	0.84	0.49–1.43	0.86	0.51–1.45	30	0.9	0.72	0.45–1.15	0.81	0.50–1.31
Negative any	131	0.8	1.00	Ref	1.00	Ref	227	1.2	1.00	Ref	1.00	Ref
Age years												
<25	46	1.4	1.00	Ref	1.00	Ref	42	1.1	1.00	Ref	1.00	Ref
25–34	56	0.8	0.58	0.36–0.92	0.58	0.37–0.92	104	1.3	1.16	0.81–1.66	1.14	0.71–1.83
35–44	35	0.6	0.46	0.27–0.79	0.48	0.29–0.81	66	1.0	0.96	0.65–1.41	0.94	0.56–1.56
≥45	16	0.3	0.41	0.22–0.75	0.31	0.17–0.57	45	1.4	1.27	0.83–1.93	1.31	0.75–2.28
Lifetime number of sex partners												
0–1	43	0.5	1.00	Ref	1.00	Ref	135	1.4	1.00	Ref	1.00	Ref
2–3	66	1.0	2.02	1.27–3.23	1.89	0.19–3.00	80	1.0	0.77	0.58–1.01	0.79	0.56–1.11
4+	44	1.2	2.41	1.49–3.91	2.23	1.36–3.64	42	1.0	0.73	0.51–1.03	0.79	0.50–1.26
Any sex in last interval												
No	30	1.6	1.00	Ref	1.00	Ref	18	0.8	1.00	Ref	1.00	Ref
Yes	123	0.7	0.48	0.31–0.74	0.44	0.28–0.70	239	1.2	1.57	1.00–2.46	1.63	1.02–2.63
New sex partner in last interval												
No	144	0.8	1.00	Ref	1.00	Ref	250	1.2	1.00	Ref	1.00	Ref
Yes	9	1.7	2.04	0.96–4.36	1.87	0.87–4.01	7	1.2	0.95	0.38–2.38	1.00	0.37–2.29

Abbreviations: CI, confidence interval; HPV, human papillomaviruses; HR, hazard ratio; HRadj, adjusted hazard ratio; Ref, reference level.

a Adjusted for positivity to the other HPV genus, age, lifetime number of sex partners, any sex in last interval, and new sex partner in last interval.

b Positive for any type of the other HPV genus at previous visit; corresponds to betapapillomavirus positivity for columns where alphapapillomavirus incidence is the outcome, and alphapapillomavirus positivity for columns where betapapillomavirus incidence is the outcome.

## Data Availability

The data are not publicly available due to ethical considerations. Participants of the Ludwig-McGill cohort study did not consent to have their data made publicly available. To access the data for research purposes, please contact Eduardo Franco (eduardo.franco@mcgill.ca) or Luisa Villa (l.villa@hc.fm.usp.br). Code for the permutation analysis is available at the Borealis repository: https://doi.org/10.5683/SP3/MQR8H9.
